# Radiation effects research foundation—a view to the future

**DOI:** 10.1093/carcin/bgaf061

**Published:** 2025-10-27

**Authors:** Joe W Gray, Andrew P Feinberg

**Affiliations:** Department of Biomedical Engineering, Knight Cancer Institute, Oregon Health and Science University, Portland, OR 97239, USA; Whiting School of Engineering, School of Medicine, and Bloomberg School of Public Health, Johns Hopkins University School of Medicine, Baltimore, MD 21205, USA; Faculty of Engineering, Tel Aviv University, Tel Aviv 6997801, Israel

**Keywords:** radiation effects research foundation, genomics, epigenomics, a-bomb survivors, epidemiology

## Abstract

**Graphical Abstract:**
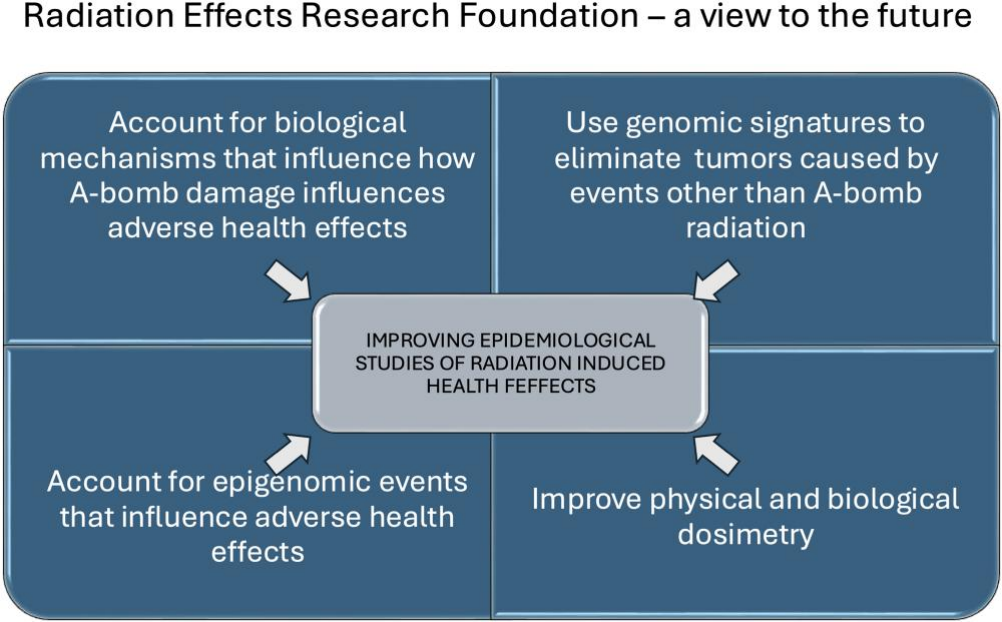

## 1. Background

The Radiation Effects Research Foundation (RERF) in Japan and its predecessor organization, the Atomic Bomb Casualty Commission (ABCC), have chronicled the effects of ionizing radiation on Japanese A bomb survivors since the founding of the ABCC in 1947—thanks to the extraordinary cooperation of the survivors and their families [1, 2]. Approximately 120 000 participants including Japanese bomb survivors and unexposed control participants from Hiroshima and Nagasaki enrolled in the RERF Life Span Study (LSS) and ∼77 000 offspring of survivors have been studied. During the course of these studies, over 2.3 million biosamples from more than 30 000 study participants have been archived. Multiple radiation-linked health effects have been uncovered by associating observed health effects with estimated radiation doses to the survivors including increased incidences of cancer, cataracts, cardiovascular disease, immune dysfunction, neurological disorders, and birth defects, particularly for individuals exposed at higher doses of radiation. This information has informed the development of radiation protection policies worldwide and in some countries, has been used as the basis for compensation for exposed individuals [3, 4]. Importantly, the work at RERF also has catalyzed the development of supporting analytical technologies at institutions outside of RERF that are now important in human health studies worldwide. For example, fluorescence *in situ* hybridization [5–7] was originally developed to enable assessment of chromosome damage in the A-bomb survivors and is now used as an important diagnostic tool in many biomedical settings. The efforts at RERF to assess radiation damage also motivated discussions in 1984 of what eventually became the human genome project [8, 9]. The information and technologies that eventually resulted have reshaped our understanding of all aspects of human biology and human health [10]. And now, after 40 years, these powerful human genome analysis ideas and tools are being deployed in current RERF studies as discussed in the following sections.

The risks in humans from radiation doses above about 100 mSv are statistically well established—especially for cancer. However, estimates of risk to exposures at lower doses have been made by extrapolating from effects of high doses to those presumed to exist at low dose. Historically, extrapolations to effects at much lower doses presume that effects are linearly related to dose, even at the lowest doses, i.e. extending to zero. This Linear No Threshold model for cancer risk has been recommended as plausible by multiple National Research Council—Biological Effects of Ionizing Radiation (BEIR) committees including the last, BEIR VII [11] and by the United Nations Scientific Committee on the Effects of Atomic Radiation, UNSCEAR [12]. The shape of the adverse health effects risk as function of dose has been explored in the LSS epidemiology study but studies so far have not been sufficiently powered to accurately define the shape of the dose response relationship at doses much below 100 mSv. In addition, important features that might influence risk have not been fully accounted for in studies to date including the possible effects of confounding or mediation by factors like epigenetic effects of stress, exposure to other sources of radiation, levels of immune and DNA damage surveillance, and exposure to agents that might influence the development of subsequent disease. As a result, the shape of the low dose response model remains unsettled and controversial [13, 14]. The BEIR VII and UNSCEAR reports noted some these limitations and suggested several areas of research intended to better inform on low dose radiation response issues [11].

More recently, a National Academies of Sciences, Engineering, and Medicine (National Academies) committee was charged by the US Congress with developing a long-term strategy for low-dose radiation research in the United States. The committee reexamined several aspects of low dose radiobiology, epidemiology, and dosimetry and made recommendations for future research directions in the United States [15]. We were members of the committee (hereafter referred to as the National Academies Committee), and believe that several committee recommendations are applicable to RERF and to other low dose radiation research programs and that, if implemented, will improve understanding of the health risks of radiation. These recommendations include applying new analytical capabilities to biosamples from A-bomb survivors and their families, using molecular methods to distinguish health effects induced by radiation from those caused by other events, improving dosimetry, and better accounting for biological factors that influence how individuals respond to radiation-induced genetic damage. We summarize here, several of these recommendations as they may apply to RERF research. While our comments are informed by the National Academies committee report and by subsequent publications, they should be not taken as recommendations for RERF by the National Academies committee or the National Academies. The views are our own.

## 2. New analysis tools

The National Academies Committee noted that enormous progress has been made in recent decades on developing powerful measurement and computational analysis tools that can be applied to new and archived biosamples [16]. These include efficient and low-cost tools that report on omic compositions (referring collectively to DNA [17], RNA [18] and protein [19]) of collections of cells from tissues, individual cells within tissues [20] and even subcellular organelles [21]. In addition, spatial analysis tools are being developed that report on the compositions, organizations and functional states of the components of intact tissues [22]. Emerging single cell analysis tools seem especially powerful for assessing heterogeneous mutations within individuals and for defining the cellular and subcellular compositions and functional states of biosamples [23]. In parallel, powerful computational tools are emerging that enable the management and biomedical interpretation of the massive amounts of data that are now being generated [24, 25]. These tools are now being used in other research programs for characterization of a broad range of normal and diseased tissues and blood and the results are being made publicly available. The data generated by these studies can serve as references for RERF research and other studies aimed at understanding how normal tissues form and function, and how exposure to a range of damaging agents changes form and function. Analysis of these data will provide guidance on how to mitigate the effects of damage [26, 27].

## 3. Disease status databases

Numerous reference databases were identified by the National Academies committee that may be useful in future RERF research to elucidate the biological changes and health effects that that arise in the individuals enrolled in RERF studies. These include GenBank, an NIH genetic sequence database of annotated collections of publicly available DNA sequences [28; the University of California, Santa Cruz’s Genome Browser [29] and Xena Browser [30], which describe the sequences, genes, and other components of the normal and aberrant genomes; the ENCODE portal [31], which informs on functional elements in human genomes including epigenomic modifications; the Catalog of Somatic Mutations in Cancer [32], which provides information on “millions of mutations across thousands of cancer types” and the UK Biobank which provides environmental, lifestyle, and genetic data on ∼500 000 participants [33, 34]. Other databases include those developed by the European 1+ million genomes initiative [35], the Sanger Cancer Genome project that reports on variations within ostensibly normal individuals [36]; RNA, DNA and protein profiles for thousands of tumors of 23 different types compiled by The Cancer Genome Atlas Project [37], single cell atlases of the normal human body [38–41], the Molecular Signatures Database which describes tens of thousands of annotated gene sets that represent well-defined biological states or processes [42], a Cardiovascular Disease Atlas compiled by the China National Center for Bioinformation [43], and the Allen Brain Cell Atlas that provides multimodal single cell information across mammalian brains [44]. Geospatial databases cataloging aspects of exposure to environmental, societal or physical events that influence human health endpoints may also be useful [45–47].

## 4. Mutational epidemiology

The ability of epidemiological studies to identify radiation-induced health effects—especially at low doses—is limited by the multi-causal nature of most instances of adverse health effects identified in such studies. For example, according to the BEIR VII report on health risks from exposure to low-dose ionizing radiation, only about two percent of cancers that arose in A-bomb survivors exposed to 100 Sv of radiation were estimated to be caused by that radiation for follow-up through 1998 [11]. When all exposure doses are considered, somewhat higher estimates for radiation induced-cancers come from the most recent follow-up of the LSS; for example,10% of solid cancer incidence (1958–2009) from Grant *et al.* Table 8 [48] and 4.8% of solid cancer death (1950–2003) from Ozasa *et al*. Table 9 [49]. In any case, most cancers in the A-bomb survivor population have causes other than exposure to radiation from the A-bombings and some might be due to the actions of multiple factors, such as smoking and radiation. Consequently, even the very large RERF studies are not sufficient to elucidate the effects of A-bomb exposures at doses much below 100 mSv with the needed precision. This limitation might be overcome, however, if adverse health effects resulting from other causes could be removed from consideration.

The National Academies Committee noted that this may become possible using information from emerging genomic studies showing that many DNA damaging agents leave characteristic damage “scars” in the genome that inform on the mechanisms by which the damage is induced. A compendium of genetic changes has been compiled by the Pan-Cancer Analysis of Whole Genomes Consortium [37] from analysis of 2658 cancer genomes from 38 cancer types and extended by a Mutographs Cancer Grand Challenge team to include information from tumors and normal tissues [50]. To date, a radiation specific signature has not been discovered. However, distinctive mutational signatures have been identified that are associated with other causative agents (e.g. smoking, aflatoxin exposure, chemotherapy, reactive oxygen species, APOBEC activity, UV exposure, and defective mismatch repair). These vary between cancers and between geographical locations. Importantly for RERF, a distinctive mutational signature of unknown cause—presumably not radiation—was shown to be present in ∼70% of cancers from Japan but not in cancers from other countries [50].

The RERF team might use information from mutational epidemiology to increase the sensitivity of their studies to radiation effects by eliminating tumors or other diseases that carry distinctive mutation spectra or other molecular indicators that indicate non-radiation etiologies. The Japan-specific mutagenic profile is particularly interesting in this regard [50]. Assessing mutagen signatures in diseased or associated normal tissues in the A-bomb survivors might have the added advantage of providing the survivors with information about possible environmental exposures other than radiation to be avoided.

A possible complementary approach to increasing sensitivity to radiation damage is to integrate “exposomics” into radiation epidemiology. Exposomics was recently defined by Miller and the Banbury Exposomics Consortium [47] to be the “integrated compilation of all physical, chemical, biological, and (psycho) social influences that impact biology”. Use of information from exposomics research might further enable RERF researchers to identify and correct for influences from other exposures that may impact endpoints being tested for association with radiation, including cancer, neurodegeneration, pulmonary disease and diabetes and to explore disease mechanisms. These might include exposures to forms of radiation other than from the A-bombs such as iatrogenic CT scans [51] and radon [52, 53]. Geospatial approaches to exposure assessment now being developed may be especially useful as models for future RERF studies [54, 55].

Concepts from mutagenic epidemiology and exposomics also might be applied during the planned RERF “Trio” study, which is searching for potential radiation induced mutations in the offspring of exposed A-bomb survivors. Identifying radiation-induced damage in the offspring may be complicated by the many spontaneously occurring mutations that are likely to occur. The “background” germline mutation rate has been estimated in other trio studies to be ∼1.2 × 10^−8^ per nucleotide per generation for SNVs [56]. Assuming 6 × 10^9^ bp of DNA per target cell, we might expect as many as 70 *de novo*, non-radiation induced mutations in each child. This number is likely to be large compared with the number of radiation-induced mutations. However, background mutations may carry distinctive mutational signatures that would allow them be excluded as radiation-induced, thereby increasing sensitivity for detection of the true radiation-induced genetic damage.

## 5. Improved radiation dosimetry

The RERF, working with collaborators, is making good progress in refining estimates of delivered dose that consider variations in exposure between anatomic sites [57, 58]. Use of these new doses should increase the sensitivity to detect radiation-induced diseases that arise in specific anatomic sites. In addition, improved biological estimates of doses might also increase the accuracy with which doses are estimated. Historically, the RERF has used measurements of the frequencies of stable chromosome aberrations as biological estimates of dose. In general, these show a strong correlation with estimated dose [7]. However, there are significant discrepancies in some individuals. Recent genome sequencing work from Li, Balmain and colleagues [59] in carcinogen treated animal models demonstrates that induced genetic mutations persist for long periods of time and manifest as cancers only after subsequent treatment with promoting agents that disrupt stromal architecture and/or immune function. This suggests that RERF might consider applying large scale, single cell sequencing to archived nucleated blood cells (or other tissues as available) and using measured mutation frequencies (after eliminating mutations with mutational spectra suggesting non-radiation causes) as a new biological estimate of dose. The utility of this approach is supported by studies showing the frequency of Glycophorin A mutations assessed using flow cytometry showed a strong correlation with estimated radiation dose [60–62]. Application of single cell sequencing might also reveal the existence of subpopulations of cells carrying disease-linked genomic aberrations that put the individual at increased risk of disease progression [63, 64].

## 6. Epigenomic events

A key concern in radiation epidemiology is that factors other than radiation-induced genetic changes may influence the incidence of health effects being tested for association with exposure to radiation. Radiation is usually considered to be the key driver of radiation dose associated adverse health effects in the A-bomb survivors and their offspring. However, many of the health effects being investigated for association with radiation exposure are also influenced by the epigenomic status of affected individuals. Epigenomic status is now understood to be altered by physical, chemical and psychosocial stress [65]. Unlike the DNA sequence *per se*, or genome, which is a static view of the genetic “words” that distinguish one person from another, the epigenome is the “grammar” that integrates environmental sensing with the DNA sequence to remember the individual’s response to environmental stress, and to pattern the response to subsequent similar stressors. The mechanisms by which epigenetic changes influence health endpoints are not fully understood. However, such changes have been found to be involved in mitochondrial adaptation to reactive oxygen species, including DNA methylation changes in the epigenomic aging clock [66]. Moreover, a DNA methylation inhibitor has been shown to attenuate hyperoxia-induced damage through changes in gene expression [67]. DNA methylation and gene expression analyses revealed consistent, time-dependent, and stable alterations related to oxidative stress response [68, 69]. Whatever the mechanism, long lived epigenomic changes have been documented in individuals suffering from post-traumatic stress disorder [70], and other neuropsychiatric diseases [71] and associated with diminished cardiovascular health [72], immune status [73], cancer [74, 75] and overall aging [66, 76]—all health effects that also might reasonably be ascribed to radiation. Importantly, epigenomic changes can be detected in blood cells or blood components [77] raising the possibility that RERF can assess such changes in the archived blood samples that they maintain.

It is important for RERF researchers to explore epigenomic changes because epigenomic changes induced by the psychosocial stress and environment changes experienced by A-bomb survivors may be loosely related to estimated radiation dose (i.e. because stress might increase with proximity to the hypocenters). If this is the case, adverse health effects thought to be caused by radiation-induced genetic changes might also have epigenomic causes. Assessment of epigenomic changes in A-bomb survivors might allow RERF to at least partially disambiguate stress-related and radiation-induced effects in epidemiological studies. Assessment of epigenomic effects may also be important in the Trio study since environmental exposures have been found to produce epigenomic changes in in sperm cells [78] that can be transmitted to offspring [79, 80].

Overall, inclusion of epigenomic information in RERF epidemiological studies may improve assessments of the effects of radiation and may identify A-bomb stress-related epigenomic changes that are themselves causally related to health effects experienced by individuals living through the aftermath of the A-bombings. Discovery of causal adverse health epigenomic events might motivate exploration of the possibility that some of these events can be mitigated using chemical or psychosocial interventions.

## 7. Biological modifiers of disease genesis

The National Academies Committee noted the remarkable advances in biological understanding of how genetic damage is transduced into disease that have occurred in recent years. A wealth of studies have revealed the mechanisms that diverse cells use to repair-induced DNA damage and have shown that that repair capacity can vary between individuals as a result of inherent genetic or induced epigenomic differences and/or by exposure to agents that upregulate basal DNA repair mechanisms. DNA profiling technologies now enable quantitative assessment of the genomic or epigenomic variations in genes involved in DNA repair pathways. In parallel, RNA and protein profiling tools allow assessment of molecular networks that control aspects of DNA repair or damage surveillance [81]. RERF might use these to explore the possibility that differences in DNA repair capacity among participants with archived biosamples may influence cancer incidence among individuals nominally exposed to the same radiation dose.

Single cell and blood component profiling tools also may enable assessment of the activities of immune surveillance and homeostatic cell control mechanisms that may prevent or delay the emergence of cancer and other diseased states. It has been known for decades, thanks to the work of Mintz [82] and Bissell [83] and later Hanahan and Coussens [84], that even advanced cancerous tissues can be “controlled” by environmental factors. More recently, Balmain et al [59] and Stratton *et al*. [50], have shown in animal models and humans that disease-linked DNA damage induced in subpopulations of cells within individuals can persist for years and often manifest as disease only when micro- and/or macroenvironments are altered in ways that enable damaged cells to escape from control. In cancer, disease promoters can be agents that alter stromal architecture [85, 86], immune contexture and/or function [87] and the levels of cytokines and other signaling molecules (e.g. from endogenous microbiota [88, 89], aging related process [90] and the brain [91–93]) that promote cancer cell growth. Importantly, advances in the measurement technologies inspired by RERF and described above may now support measurement of biomarkers that report on disease control features in ways that will reveal the mechanisms that become dysregulated during disease progression [94]. This, in turn, could benefit the survivor community by suggesting therapeutic strategies to counter the loss of control.

## 8. Conclusion

Remarkable advances have been made in recent years in cell and molecular measurement technologies, development of data bases that describe molecular characteristics of normal and diseased states, and mechanistic understanding of how damaged cells lose contextual control and function to emerge as diseased tissues. The RERF is now poised to deploy these new tools and knowledge to improve understanding of how radiation damage leads to diverse diseases and to identify strategies to mitigate the health effects of radiation exposure. In so doing the RERF will further its efforts to understand “…the medical effects of radiation and associated diseases in humans for peaceful purposes, contributing to the maintenance of the health and welfare of atomic bomb survivors and enhancing the health of all humankind”.

## Data Availability

No new data were generated or analysed in support of this commentary.
